# Early Structural and Functional Defects in Synapses and Myelinated Axons in Stratum Lacunosum Moleculare in Two Preclinical Models for Tauopathy

**DOI:** 10.1371/journal.pone.0087605

**Published:** 2014-02-03

**Authors:** Hervé Maurin, Seon-Ah Chong, Igor Kraev, Heather Davies, Anna Kremer, Claire Marie Seymour, Benoit Lechat, Tomasz Jaworski, Peter Borghgraef, Herman Devijver, Geert Callewaert, Michael G. Stewart, Fred Van Leuven

**Affiliations:** 1 Experimental Genetics Group - LEGTEGG, Dept. Human Genetics, Leuven, Belgium; 2 Dept. Life Sciences, Open University, Milton Keynes, United Kingdom; 3 Research Group Neurodegeneration, KULAK, Kortrijk, Belgium; Universidad de Sevilla, Spain

## Abstract

The stratum lacunosum moleculare (SLM) is the connection hub between entorhinal cortex and hippocampus, two brain regions that are most vulnerable in Alzheimer’s disease. We recently identified a specific synaptic deficit of Nectin-3 in transgenic models for tauopathy. Here we defined cognitive impairment and electrophysiological problems in the SLM of Tau.P301L mice, which corroborated the structural defects in synapses and dendritic spines. Reduced diffusion of DiI from the ERC to the hippocampus indicated defective myelinated axonal pathways. Ultrastructurally, myelinated axons in the temporoammonic pathway (TA) that connects ERC to CA1 were damaged in Tau.P301L mice at young age. Unexpectedly, the myelin defects were even more severe in bigenic biGT mice that co-express GSK3β with Tau.P301L in neurons. Combined, our data demonstrate that neuronal expression of protein Tau profoundly affected the functional and structural organization of the entorhinal-hippocampal complex, in particular synapses and myelinated axons in the SLM. White matter pathology deserves further attention in patients suffering from tauopathy and Alzheimer’s disease.

## Introduction

Deteriorating intellectual and mental faculties are all but the most feared in the elderly in our ageing society. Despite extensive fundamental and clinical investigations and trials, the lack of preventive or effective treatment of neurodegenerative disorders accounts for the increasing number of people suffering from dementia, in particular Alzheimer’s Disease (AD) [1]. Besides the well-known defects in gray matter, also problems with white matter are recognized in AD. These include structural defects in, with partial to extensive loss of myelin sheaths, inflicting axons with microstructural changes that remain largely to be defined [2]–[9]. Interestingly, myelin defects observed in the perforant pathway (PP) strengthened the hypothesis that pathologically altered anatomical pathways contribute importantly to mental disease [6], [7], [10].

The entorhinal cortex (ERC) is considered an early and most vulnerable region in AD-brain. Accumulation of phosphorylated protein tau in all its layers precedes its progressive accumulation in the hippocampal formation [11], [12]. ERC and hippocampus are intimately involved in various forms of learning and memory that are affected in AD, which make entorhinal-hippocampal projections of prime interest, both functionally and in relation to the proposed ‘spreading’ or sub-regional progression of tauopathy [13]–[17].

ERC-neurons project to the dentate gyrus (DG) and CA1 hippocampal subfields by the perforant (PP) and temporoammonic pathway (TA) that originate in ERC layers II and III, respectively [18], [19]. The TA pathway links ERC to CA1 pyramidal neurons by synapsing onto distal parts of apical dendrites within the stratum lacunosum moleculare (SLM) [20]. Although most afferents in CA1 SLM constitute the TA with afferents from ERC layers II and III, also ERC layers V and VI contribute [19]. Early in AD, the apical dendrites in SLM develop phospho-tau positive dilatations resulting in degeneration, termed dendritic amputation [21].

Attention has been focused mainly on PP that targets DG, while TA remained relatively neglected, even so far that the term PP usually also refers to TA. Nevertheless, TA was defined of prime importance for long-term memory consolidation and for intermediate-term memory retention and retrieval [22]–[24].

Recently we reported that expression of Nectin-3, an important cell adhesion molecule (CAM) was compromised in the CA1 SLM of two transgenic models for tauopathy, carrying Tau.P301L without or with GSK3β [25]. We now tested the hypothesis that early phases of tauopathy impact the structure and functions of synapses and dendritic spines in the ERC-hippocampal formation of the Tau.P301L and biGT models. The combined data demonstrated that tauopathy provoked early and profound impairments of the functional and structural organization of the hippocampal circuits that link ERC to the hippocampus proper, in particular synapses and myelinated axons within the SLM.

## Materials and Methods

### Ethical statement

All experiments were performed in accordance with regional, national and European regulations concerning animal welfare and animal experimentation, and were authorized and supervised by the University animal welfare commission (Ethische Commissie Dierenwelzijn, KULeuven).

### Mouse models

Generation and validation of Tau.P301L mice and bigenic Tau.P301LxGSK3β.S9A mice (denoted biGT) were reported [26], [27]. Analysis of dendritic spines was performed in crosses with Thy1-YFP mice, as described [28], [29]. All offspring was genotyped for the respective transgenes by PCR and qPCR on DNA isolated from tail biopsies [25], [27], [29].

Intracerebral injection of AAV-Tau.4R in the entorhinal cortex was as described [30], [31]. Briefly, AAV-Tau.4R virus (10^8^ transducing unit) was stereotactically injected at coordinates posterior 4.72 mm, lateral 3.25 mm, ventral 3.5 mm relative to Bregma, and injected mice were analyzed 1 month post-injection.

### Behavior

**Passive Inhibitory avoidance task (PIA)** was performed in a two-chambered inner box comprising lit and dark sections separated by a trap-door, all placed inside a larger sound-tight box. For conditioning mice were placed in the lit section and after 10 sec the trap-door was opened to allow entrance in the dark section where they received an electric footshock (0.5 mA; 2 sec) after 2 sec. The mice were kept 15 sec in the dark compartment before being returned to the home cage. Retention was assessed after 24 hours by placing the mouse in the lit section and measuring the latency as the time that elapsed before entry into the dark compartment [27].

**Novel Object recognition task (NORT)** was essentially as described [32]–[34]. Briefly, mice were habituated for 10 min in the perspex open-field box dimly illuminated from below. The next day, mice were observed in the same box for 8 min in the presence of two identical objects (A). The time was recorded that the mice explored both objects with criterion that the snout was directed towards and close to the object (less than 1 cm). The exploration time was recorded as the measure for explorative behavior. The same day, 4 hours later an 8 min retention trial was performed by placing the mouse in the box with one old (A) and a novel object (B). The time that the animal spent exploring each object (tA, tB) was recorded. The relative retention index (RI) was defined as the time spent exploring novel object (B) over the time spent exploring both objects, i.e. tB/(tA+tB)×100.

### Biochemical analysis

Mice were sacrificed by decapitation and the brain rapidly removed, hippocampi promptly dissected and homogenized as described [25]. Proteins were denatured, reduced and separated on 10% Tris-Glycine SDS-PAGE gels (Anamed, Germany). After transfer, nitrocellulose membranes were probed with primary antibodies specific for protein tau and tubulin as described [25] and for CNPase (Millipore - MAB326) as marker for myelin.

### Immunohistochemistry

Mice were anesthetized (Nembutal; 100 mg/kg, i.p.) before transcardiac perfusion with ice-cold saline (4 ml/min, 2 min). Brains were quickly removed to allow an overnight fixation in 4%paraformaldehyde in PBS at 4°C. Brains were stored in 0.1% sodium azide in PBS at 4°C until vibratome 40 µm thick sections were collected.

Immunohistochemistry was performed essentially as described [26], [30]. For CNPase (Millipore - MAB326) an antigen retrieval step was necessary. Briefly, sections were mounted on silanized glasses, dried for one hour at 37°C and incubated in a decloaking chamber in proprietary decloaking solution (Diva, Biocare Medical). After rinsing in PBS, sections were pretreated for 15 min in a 1.5% H_2_O_2_ in 50% methanol/PBS to suppress endogenous peroxidase activity. Next, blocking of nonspecific binding sites was by incubation in blocking buffer (10% fetal calf serum, 0.1% Triton X-100 in PBS). The sections were incubated at 4°C overnight with primary monoclonal antibodies: HT7 and AT100 (Innogenetics, Gent), AT180 and AT8 (Thermo Scientific), appropriately diluted in blocking buffer.

After rinsing in 0.1% Triton X-100 in PBS, sections were incubated for 1 hr with the appropriate secondary antibodies (1∶500 in blocking buffer). Alternatively as required, sections were incubated with avidin-biotin complex (Vector Laboratories - Vectastain ABC Elite kit) for 30 min, rinsed in PBS and incubated for 5 min in 50 mM Tris-HCl (pH7.6). Enzymatic staining was performed using a solution of 3,3′-diaminobenzidine (0.5 mg/ml), 0.3% H_2_O_2_ in 50 mM Tris-HCl (pH 7.6). Hematoxylin counterstaining was prior to dehydration in a graded ethanol series. After two washes in 100% xylol, the sections were mounted with DePeX. Quantification of IHC staining was performed blindly, assigning ascending scores from 0 to 2, for negative to intense staining, respectively.

### CA1 SLM Tracking with DiI crystals implanted in the entorhinal cortex

Mice were anesthetized (Nembutal; 100 mg/kg, i.p.) before transcardiac perfusion with ice-cold saline (4 ml/min, 2 min) followed by 4% paraformaldehyde perfusion (4 ml/min, 10 min). Brains were removed and post-fixed by immersion in 4% paraformaldehyde at 4°C for 3 days. A single crystal of the carbocyanine 1,1′-dioctadecyl-3,3,3′,3′-tetramethylindocarbocyanine perchlorate (DiI, D-3911, Invitrogen) [35], [36] was implanted in the entorhinal cortex at coordinates: 5 mm posterior to bregma, 2.8 mm lateral to midline and 3 mm ventral, determined using a Vernier caliper. Brains were stored in 4% paraformaldehyde at room temperature for 14 months, to allow the dye to diffuse. After rinsing the brains in PBS, vibratome sections (40 µm) were cut and mounted on glass slides under coverslip, in 65% glycerol in PBS [37]. Microscopic images were collected (Leica microscope with appropriate filters). Higher magnifications were acquired by confocal microscopy (Olympus Fluoview 1000). Quantification was performed with dedicated software (Qwin, Leica).

### CMC myelin tissue staining

Sagittal free-floating sections (40 µm) from the same brains used for DiI tracings were incubated for myelin staining with 3-(4-aminophenyl)-2H-chromen-2-one (CMC) [38]. Briefly, sections were incubated in 1% H_2_O_2_ in 10% Triton X-100 (in PBS) for 10 minutes, before 30 min incubation at room temperature with 100 mM CMC (#9869 Matrix Scientific) in 1% DMSO diluted in PBS. Sections were rinsed with PBS three times for 5 minutes before microscopic analysis.

### Dendritic spines

Dendritic spines were analyzed as published [29] by confocal microscopy (Olympus Fluoview 1000) in the region of interest, CA1 SLM in brain of Tau.P301LxYFP bigenic mice. In total 9 independent images per mouse from 5 mice per genotype were acquired and analyzed on confocal z-stacks (0.3 µm) without post-processing. Spines were defined visually and counted manually. Spine density is expressed as the number of spines per 10 µm length of dendrite. Spine maturation index is defined as the ratio of mushroom spines to all other spine types, with mushroom spines defined by the spine head at least twice as wide as the spine neck [29].

### Electron microscopy-ultrastructure analysis

Mice were anesthetized (Nembutal; 100 mg/kg, i.p.) before transcardiac perfusion with ice-cold saline (4 ml/min, 2 min) followed by perfusion with Karnovsky fixative for 10 minutes. Brains were quickly removed and fixed in Karnovsky solution for 5 days. Thick vibratome sections (300 µm) were incubated in 1% OsO4 solution at room temperature prior to dehydration in a graded ethanol series, and finally embedded in agar100 epoxy resin. Regions of interest were trimmed and 70 nm serial sections were cut using a diamond knife (Reichert ultramicrotome). Sections were collected on formvar-coated single slot grids.

The CA1 SLM region was explored for spines at 4000x magnification (Jeol JEM 1400 microscope) and for myelin structural analysis at 25000x magnification (Jeol JEM 1010 microscope). Spine reconstruction was performed based on ribbons composed of at least 25 serial sections (70 nm thick), and from 10 different areas within the CA1 SLM. Serial sections were aligned (sEM software), and spine and PSD contours were delineated manually (Reconstruct software, http://synapses.clm.utexas.edu), allowing 3D reconstruction of each structure of interest. Spine density was assessed using the optical dissector for mushroom, thin spines and shaft synapses. Spine volume, PSD parameters and spine length were calculated by the same software. Mushroom, thin and stubby spines were categorized following similar criteria [39], with threshold set to 250 nm for spine heads to distinguish mushroom from thin spines.

Myelin structure in CA1 SLM and TA pathway was analyzed in at least 20 image per mouse, which in total contained more than 300 axons representing between 1.5 to 2 mm of myelin sheath. The G-ratio of inner axonal diameter over the total outer diameter including myelin was measured on the same myelin axonal structures.

### Electrophysiology

Acute hippocampal slices were prepared as described [40]. Briefly, brains were quickly removed and cut horizontally into 250 µm slices in ice-cold artificial cerebrospinal fluid (ACSF) containing (in mM) 125 NaCl, 2.5 KCl, 2 CaCl_2_, 1 MgCl_2_, 1.25 KH_2_PO_4_, 25 NaHCO_3_, and 25 glucose, saturated with 95% O_2_/5% CO_2_ (pH 7.2–7.3). The CA3 and DG regions were sectioned to separate the SLM layer of the CA1 region from the trisynaptic circuit. Sectioned slices were incubated in a recovery chamber containing oxygenated ACSF at room temperature for at least two hour before recording.

Extracellular stimulation and recordings were performed using a multi-electrode array (MEA) (Multi Channel Systems, Martinsried, Germany) as described [40]. Sections were continuously perfused with oxygenated ACSF (3 ml/min) at 31°C containing 20 µM bicuculline to block fast inhibitory transmission. TA was stimulated by electrodes positioned under SLM near the hippocampal fissure. Evoked field excitatory postsynaptic potentials (fEPSPs) were monitored [41], [42]. Input/Output curves were generated by collecting responses to a series of increasing biphasic voltage pulses of 200 µs duration. The stimulus intensity used in subsequent recordings was set to evoke fEPSP of 50% of maximal amplitude. Short-term plasticity was addressed before LTP induction. Paired-pulse facilitation (PPF) was evaluated using two stimulations at inter-pulse intervals (IPI) between 20 and 500 msec. Short-term depression was assessed by measuring fEPSP amplitude in response to a train of pulses at 100 Hz. LTP was induced, after recording a stable baseline for 15 min, by 4 trains of high frequency stimulation (HFS) at 100 Hz for 1 sec with 20 sec intervals. At the end of the recordings, the mGluR2 agonist 2-(2,3-dicarboxycyclopropyl)glycine (DCG IV, Tocris, UK), was routinely added (3 µM final concentration) to verify TA responses [42], [43]. All MEA data were collected at 10 kHz sampling frequency and 1100 amplifier gain. fEPSPs were recorded and analyzed by dedicated software (MC_Rack & MC_Data Tool software, Multi Channel Systems, Germany).

### Statistical analysis

Statistical analysis was performed using dedicated software (GraphPad Prism v5.03; San Diego, CA). Data were plotted as median (for scoring) or mean±SEM. Data-sets were analyzed either by Student’s t-test (unpaired, two-tailed) or One-way ANOVA, followed by Bonferroni *post hoc* test as indicated in the figure legends. For electrophysiological experiments, data (mean ± SEM) from transgenic and wild-type mice were compared by One- or Two-way ANOVA (Origin 8.0, Origin Lab). Statistical significance was defined as p<0.05.

## Results

Most recently, we demonstrated that neuronal expression of human protein Tau, either wild-type or mutant, engendered pronounced and specific reduction of Nectin-3, the major synaptic cell adhesion molecule (CAM) [25]. The defect was most pronounced in the SLM, the connection hub between ERC and hippocampus proper, two brain regions at the center of attention in clinical and experimental studies in AD. The ERC projects by myelinated axons forming the TA to the CA1 SLM by excitatory synapses on distal sections of the apical dendrites of the CA1 pyramidal neurons.

Here we studied the early functional and structural repercussions of tauopathy in the preclinical models, denoted Tau.P301L and biGT mice, from early age (3 months) when significant Nectin-3 expression was already lost, prior to the more classical tangle tauopathy [25]. We investigated the surmised relation of lacking Nectin-3 to impaired cognition in Tau.P301L mice. We subsequently measured density and morphology of dendritic spines, as well as their post-synaptic densities (PSD), all prime structural parameters of synaptic plasticity, essential for learning and memory [29], [44], [45].

We went on to define progression of Tauopathy in the Tau.P301L mice and comparatively also in the other validated model for tauopathy: bigenic Tau.P301LxGSK3β.S9A mice, denoted biGT. Both models present with tauopathy progressing with age but on different time-scales: age 7–11 months for Tau.P301L mice, and 10–18 months for biGT mice [26], [27]. Besides age of onset, both models present regional differences in their intensity of eventual tauopathy [26], [27], [29], [46]–[50]. Nevertheless, the terminal phase was very similar in both models, despite the age difference, by combination of clinical indices that progress rapidly (2–3 weeks) leading to death: reduction in bodyweight, increased clasping of hind- and then fore-limbs, hyperkyphosis and inactivity, upper airway dysfunction caused by defective brainstem circuits [46], [50], [51]. The endpoint was invariably precocious death, for Tau.P301L mice mostly between age 8–12 months (average 9 months) without survivors beyond age 12 months [29]. The age of death of biGT mice was less sharply defined, ranging from 10 to 22 months [26], [27], [46], [50], [51]. The mechanistic contributions of GSK3β in the biGT model to the delay in tauopathy and to clinical phenotype are subject of ongoing studies.

### Cognitive defects in ageing tau.P301L mice

Tau.P301L mice were analyzed for cognitive capacity in two tasks of different complexity: the novel object recognition task (NORT) and passive inhibitory avoidance (PIA). Both tasks do not depend heavily on physical and motor abilities, which become compromised in Tau.P301L mice, as in most Tau transgenic mice [26]. Moreover, the NORT, as performed, measures predominantly hippocampal plasticity [52]. PIA is a more complex task, involving not only the hippocampus but also amygdala and cortical regions [53].

**Figure 1:**
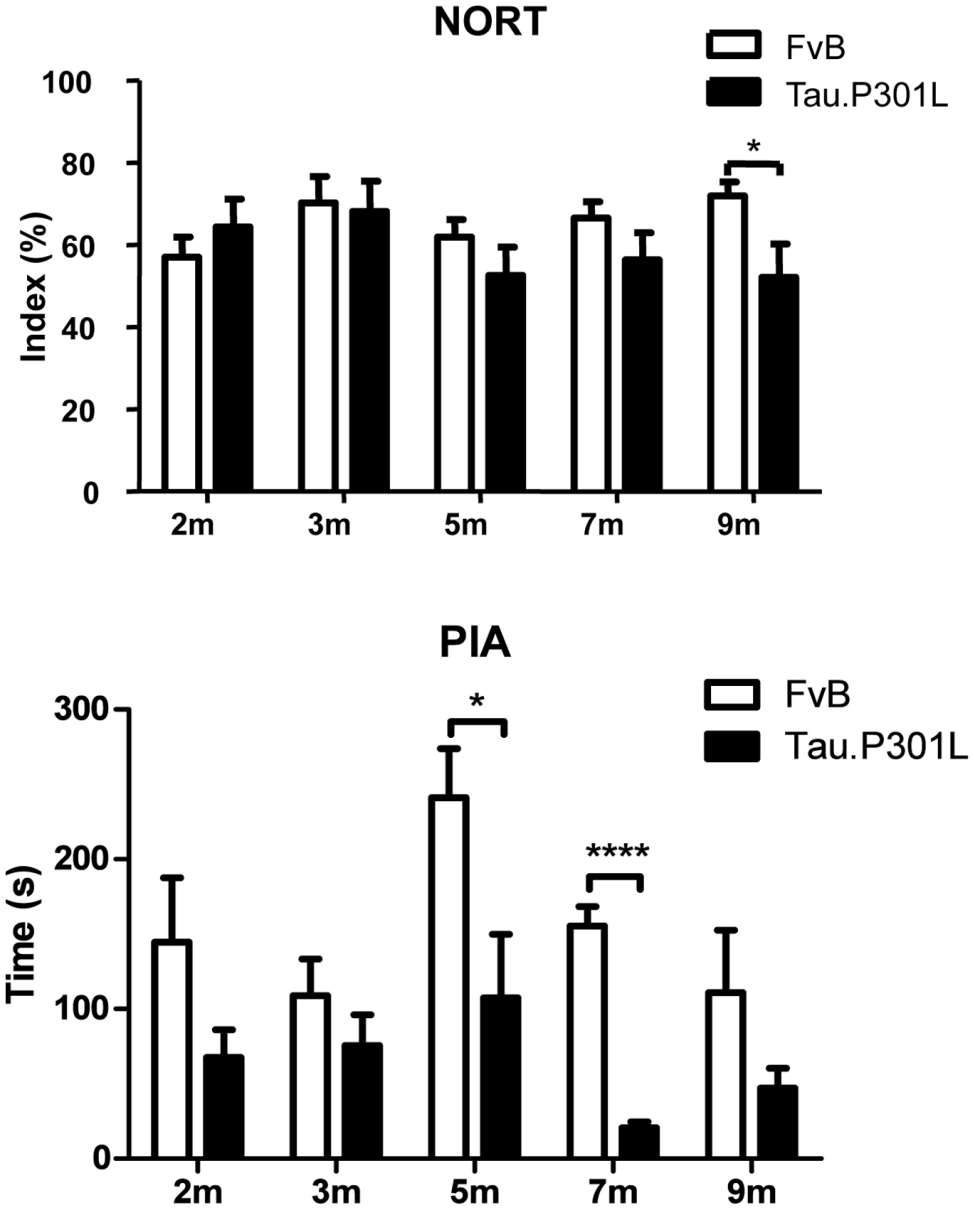
Defective cognition of Tau.P301L mice at different ages. Analysis by novel object recognition (ORT) (upper panel) and passive inhibitory avoidance (PIA) task (lower panel) of Tau.P301L mice at age 2 to 9 months compared to age-matched wild-type FvB mice. Student t-Test, unpaired, two-tailed, n = 8 per age group. *p<0.05, ****p<0.0001.

NORT demonstrated cognitive defects in ageing Tau.P301L mice that approached but were not yet terminal and still devoid of clasping (Fig. 1). Conversely, the PIA task revealed cognitive defects in Tau.P301L mice already at young age. Some variation with age was attributed to the complexity of the task and the known involvement of different brain regions, differently attained by the progressing tauopathy (Fig. 1). Of note, the molecular characteristics of Tau.P301L differ in different brain regions and change with age in terms of phosphorylation and aggregation [50].

Most interesting, the behavioral defects in Tau.P301L mice correlated with defective Nectin-3 levels in CA1 SLM [25]. We therefore engaged to approach functional and brain-structural aspects, first in Tau.P301L mice, and then extended the analysis to biGT mice, to define pathological contributions of GSK3β [27], [29], [31].

### Dendritic spines in CA1 SLM of Tau.P301L mice

**Figure 2:**
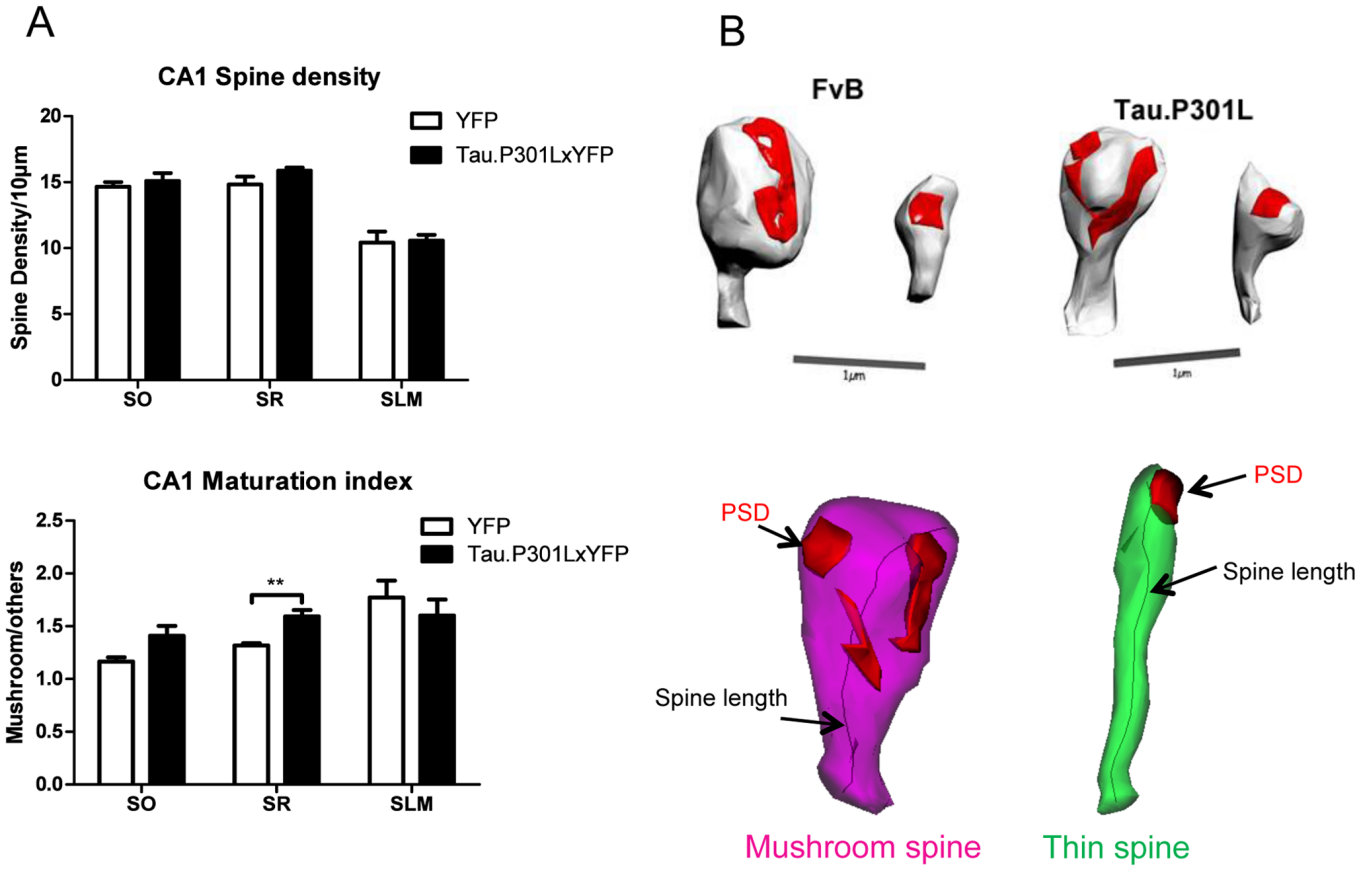
Analysis of dendritic spines in SLM of Tau.P301L mice. A. Dendritic spines in Tau.P301Lx YFP bigenic mice (n = 5) in different brain regions: stratum oriens (SO), stratum radiatum (SR), CA1 SLM as indicated in the captions. Upper panel: spine density is expressed as number of spines per 10 µm of dendritic shaft. Statistical analysis by Student t-Test, unpaired, two-tailed: SO, p = 0.066; SR, p = 0.0068; SLM, p = 0.47. Lower panel: spines maturation ratio, expressed as number of mushroom spines to all spines. Statistical analysis by Student t-Test, unpaired, two-tailed: SO, p = 0.58; SR, p = 0.17; SLM, p = 0.87. B. Representative reconstructions of dendritic spines in wild-type FvB and Tau.P301L mice.

The cognitive analysis implicated that synaptic plasticity was already affected in young Tau.P301L mice, which was corroborated by analysis of dendritic spines in the SLM [29]. Spine density was, however, not affected in sub-regions SO and SR of the hippocampal formation of Tau.P301LxYFP mice up to age 6 months (Fig. 2A). We concluded that early stages of tauopathy did not negatively affect the development and maintenance of dendritic spines.

The spine maturation index, defined as the ratio between the density of mushroom spines to all other spines, is an index for active and functional synapses [29], [54]. Unexpectedly, in young Tau.P301L mice the spine maturation index was increased, significantly in stratum radiatum (SR), and borderline in stratum oriens (SO) (p = 0.0068 and 0.0646, respectively; Student t-test unpaired, two-tailed; Fig. 2A). In contrast, the spine maturation index tended to be decreased in the SLM of Tau.P301L mice (Fig. 2A).

The morphology of mushroom and thin spines was analyzed in ultrastructural detail by 3D reconstruction of electron micrographs [39], [55]. The results confirmed that the overall density of spines was not significantly affected in young Tau.P301L mice relative to wild-type mice, matched for age, gender and genetic background (Table 1). Moreover, the ultrastructural data on Tau.P301L mice corroborated the foregoing spines dataset obtained by confocal microscopy in Tau.P301LxYFP mice: the density of mushroom spines in SLM was significantly lower in young Tau.P301L mice than in wild-type mice (age 4 months; p = 0.0037; Student t-Test unpaired, two-tailed; Table 1). The volume of mushroom spines was not affected in Tau.P301L mice, while a trend of decreased volume of thin spines was noted (Table 1). The third parameter analyzed, the mean overall length of mushroom and thin spines within the CA1 SLM was similar in both genotypes.

**Table 1:** Parameters of dendritic spines in CA1 SLM of wild-type FvB and Tau.P301L mice.

	Spine density					Spine volume		Spine length		PSD volume		PSD area	
	(spines/100 µm^3^)					(µm^3^)		(µm)		(µm^3^)		(µm^2^)	
	Mush-room	Thin	Shaft	All	Maturationindex	Mush-room	Thin	Mush-room	Thin	Mush-room	Thin	Mush-room	Thin
Tau	23.36	30.18	8.15	61.68	0.7921	0.2115	0.0505	1.326	0.9957	0.01148	0.00369	0.1646	0.05315
P301L	+/−	+/−	+/−	+/−	+/−	+/−	+/−	+/−	+/−	+/−	+/−	+/−	+/−
	0.51	3.35	1.50	4.44	0.0815	0.0077	0.0030	0.023	0.0786	0.00031	0.00004	0.0048	0.00093
wild	26.51	29.65	11.24	67.40	0.9003	0.2161	0.0622	1.343	1.006	0.00997	0.00405	0.1419	0.05798
type	+/−	+/−	+/−	+/−	+/−	+/−	+/−	+/−	+/−	+/−	+/−	+/−	+/−
FvB	0.08	1.79	2.03	2.37	0.0517	0.0141	0.0050	0.067	0.0519	0.00016	0.00033	0.0032	0.00483
p	0.004	0.896	0.286	0.319	0.325	0.787	0.114	0.821	0.915	0.014	0.342	0.018	0.381

Interestingly, both the area and the volume of the post-synaptic density (PSD) were larger in mushroom spines in the SLM of Tau.P301L mice compared to wild-type mice (p = 0.0177 and p = 0.0138, respectively; t-Test; Table 1). Of note, a compensatory increase in PSD area was proposed based on higher than normal levels of the PSD95 protein in brain of AD patients [56].

We concluded that dendritic spines were altered in the early stages of progressive tauopathy in pre-clinical Tau.P301L mice. These defects correlated with defective Nectin-3 levels, and fit the context of their cognitive defects, all predating the development of classical tauopathy defined by large intra-neuronal tau aggregates. We therefore went on to characterize the status of phosphorylation of protein tau, focusing mainly on the ERC and SLM regions, and compared Tau.P301L to biGT mice in an attempt to define the contributions of GSK3β.

### Time-line of tauopathy parameters in hippocampal sub-regions of Tau.P301L and biGT mice

In the hippocampal formation, the ERC communicates with the hippocampus proper by the PP and TA projections that classically originate in ERC layers II and III respectively [19], [57], [58]. In AD, the loss of neurons starts and becomes most extensive in the ERC and CA1 [11], [59]–[61]. The tri-synaptic pathway constitutes a major feature of the hippocampal circuitry that is essential for learning and memory (L&M), but also prime suspect for contributions to spreading of pathology and cognitive defects in AD [11], [19], [60].

Tau pathology was analyzed by immunohistochemistry, first for phospho-epitope pT231 defined by antibody AT180, because it is one of the earliest markers for tauopathy in AD, and widely used as clinical biomarker in CSF for early diagnosis [62]–[64]. We then supplemented phospho-epitope pS199/S202-Tau define by antibody AT8 as another classical marker in general use for post-mortem staging of brain tauopathy in AD, although not for as CSF-biomarker in clinical diagnosis [11], [12], [60].

#### ERC

**Figure 3:**
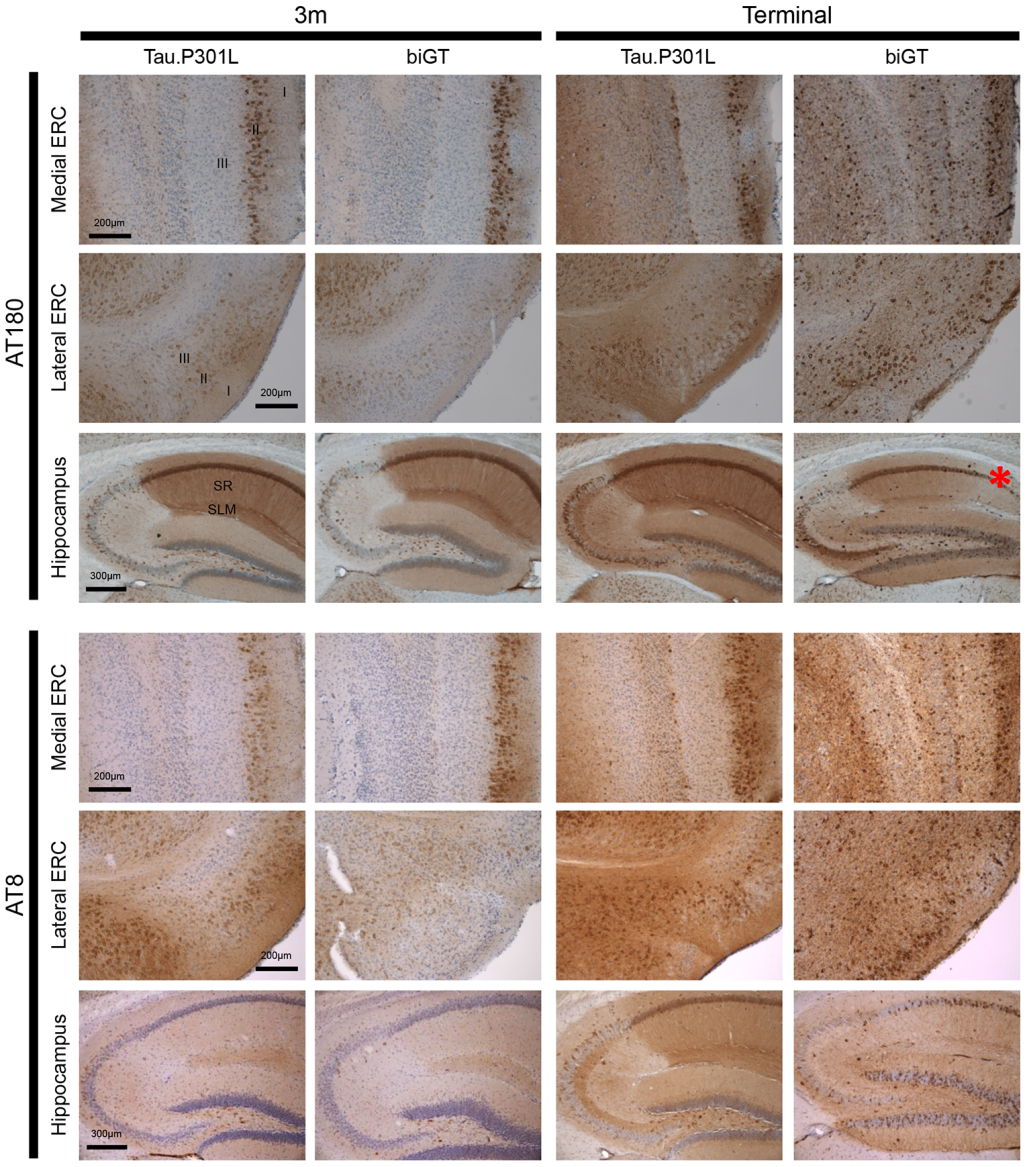
Timeline of phospho-Tau in hippocampal formation of Tau.P301L and biGT mice. IHC for pT231-Tau (AT180) and pS199–202 (AT8) in the sub-regions of the hippocampal formation of Tau.P301L and biGT mice at 3 months of age and at terminal stage (see text for details). Red asterisk marks CA1 pyramidal layer degeneration. Images are representative for the median levels of AT180 and AT8 staining (n = 6–8 mice per age group). Denoted are layers I, II and III of the entorhinal cortex and CA1 hippocampal sub-regions stratum radiatum (SR) and stratum lacunosum moleculare (SLM).

In both genotypes, and already at young age (3 months) the pT231-Tau epitope was evident in many neurons in layer II of the medial ERC (Fig. 3, upper left). In contrast, only some neurons were immunoreactive in layer III of the lateral ERC, and even less in the medial ERC (Fig. 3). With age, AT180 immunoreactivity progressed in all ERC layers and all sub-regions in both Tau.P301L and biGT mice, without marked differences with the genotype until age 9 months, before mice entered the terminal phase.

In terminal Tau.P301L mice, the ERC was mildly marked by IHC with AT180, in contrast to terminal biGT mice in which the ERC was heavily loaded with AT180 positive neurons (Fig. 3, upper right). IHC with antibody AT8 yielded similar labeling to AT180 in young Tau.P301L and biGT mice, but much more positive neurons that also stained more densely in ERC and hippocampus of terminal biGT mice than in terminal Tau.P301L mice (Fig. 3).

We concluded that not the terminal phenotype, but old age was the more important determinant for tauopathy in the ERC of the Tau.P301L and biGT mice, which is reminiscent of the fact that ageing is the most important determinant in AD.

#### CA1 and SLM

**Figure 4:**
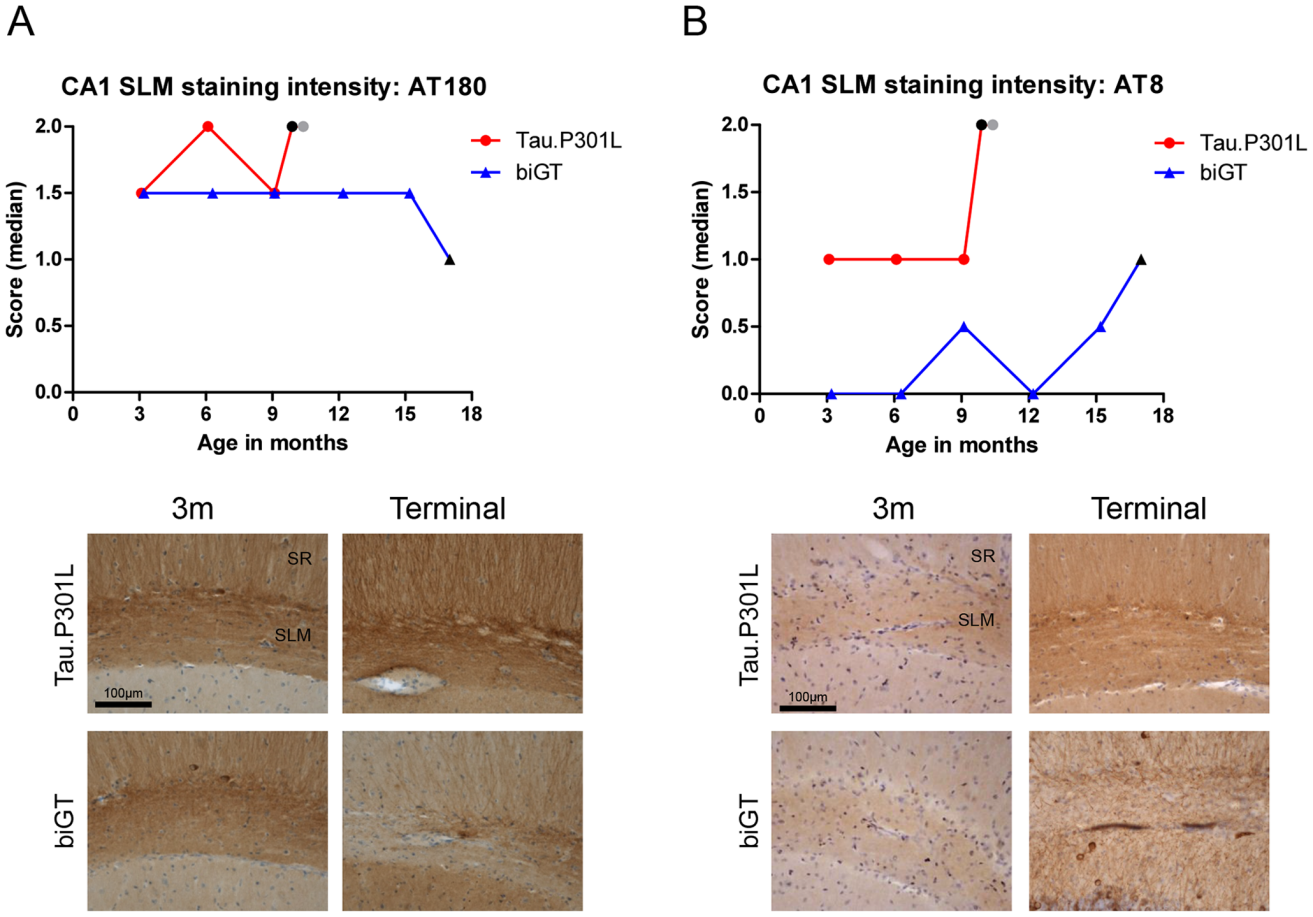
Timeline of phospho-Tau in the CA1 SLM of Tau.P301L and biGT mice. IHC for AT180 (A) and AT8 (B) staining in CA1 SLM region of Tau.P301L and biGT mice at ages 3, 6 and 9 months and terminal. Data are represented as median intensity score for immunoreactivity with AT180 (A) and AT8 (B) in CA1 SLM. Grey symbols denote mice with clasping and black symbols denote terminal mice (see text for details)(n = 6–8 mice per age group). CA1 hippocampal sub-regions stratum radiatum (SR) and stratum lacunosum molecular (SLM) are marked.

Pyramidal neurons in CA1 of Tau.P301L and biGT mice stained uniformly positive by IHC with AT180, demonstrating that protein Tau.P301L became readily and similarly phosphorylated at the pT231 epitope in both genotypes, and already at young age. Neither age, nor terminal stage markedly affected the phosphorylation of protein Tau at T231 (Fig. 3, Fig. 4A). Of note, CA1 neurons in wild-type FvB mice remained completely devoid of immunoreaction for this epitope, even at old age.

In the SLM, AT180 immunoreactive neuronal processes in both Tau.P301L and biGT mice were evident already at young age, without marked age-dependent changes (Fig. 3, Fig. 4A). Interestingly, the reduction in AT180 immunoreactivity in terminal biGT mice was fully accounted for by the extensive neurodegeneration in the CA1 sub-region bordering the subiculum (Fig. 3, red asterisk; Fig. 4A).

We similarly analyzed phospho-epitope pS199/S202-Tau defined by antibody AT8 for evolution with age. AT8 staining was less intense than AT180 in SLM of Tau.P301L mice, except in terminal mice (Fig. 4). Unexpected was the lesser AT8 reactivity in the SLM of biGT mice relative to age-matched Tau.P301L mice (Fig. 4). Clearly, neuronal tracts in the SLM of ageing biGT mice remained longer devoid of the AT8 epitope, which became more intense only in old, terminal biGT mice (13–18 months). Even then, AT8 immunoreaction remained less pronounced than in terminal, but younger Tau.P301L mice (Fig. 4B).

The combined data demonstrated that the progression of phosphorylation of protein Tau leading to tauopathy was particularly evident in the medial and lateral ERC and in the hippocampal sub-regions, including the SLM with its myelinated axons from ERC. We concluded that the tauopathy in the SLM differentiated Tau.P301L and biGT mice with respect to two major phospho-epitopes, AT8 and AT180. Nevertheless, because of inherent higher GSK3β activity in biGT mice, the anticipated higher levels of AT180 and AT8 reflecting more extensive phosphorylation of protein Tau by GSK3β was not substantiated by the experimental observations.

### Electrophysiology of synapses in SLM

The unexpected negative contribution of GSK3β in biGT mice to the levels of phospho-epitopes and to the delayed tauopathy, would be substantiated by biochemical examination, which is evidently not possible for the tiny sub-regions of the hippocampus. Therefore, we engaged to define functional and structural parameters and defects inflicted by tauopathy to the hippocampal formation, concentrating in particular on the SLM sub-region.

**Figure 5:**
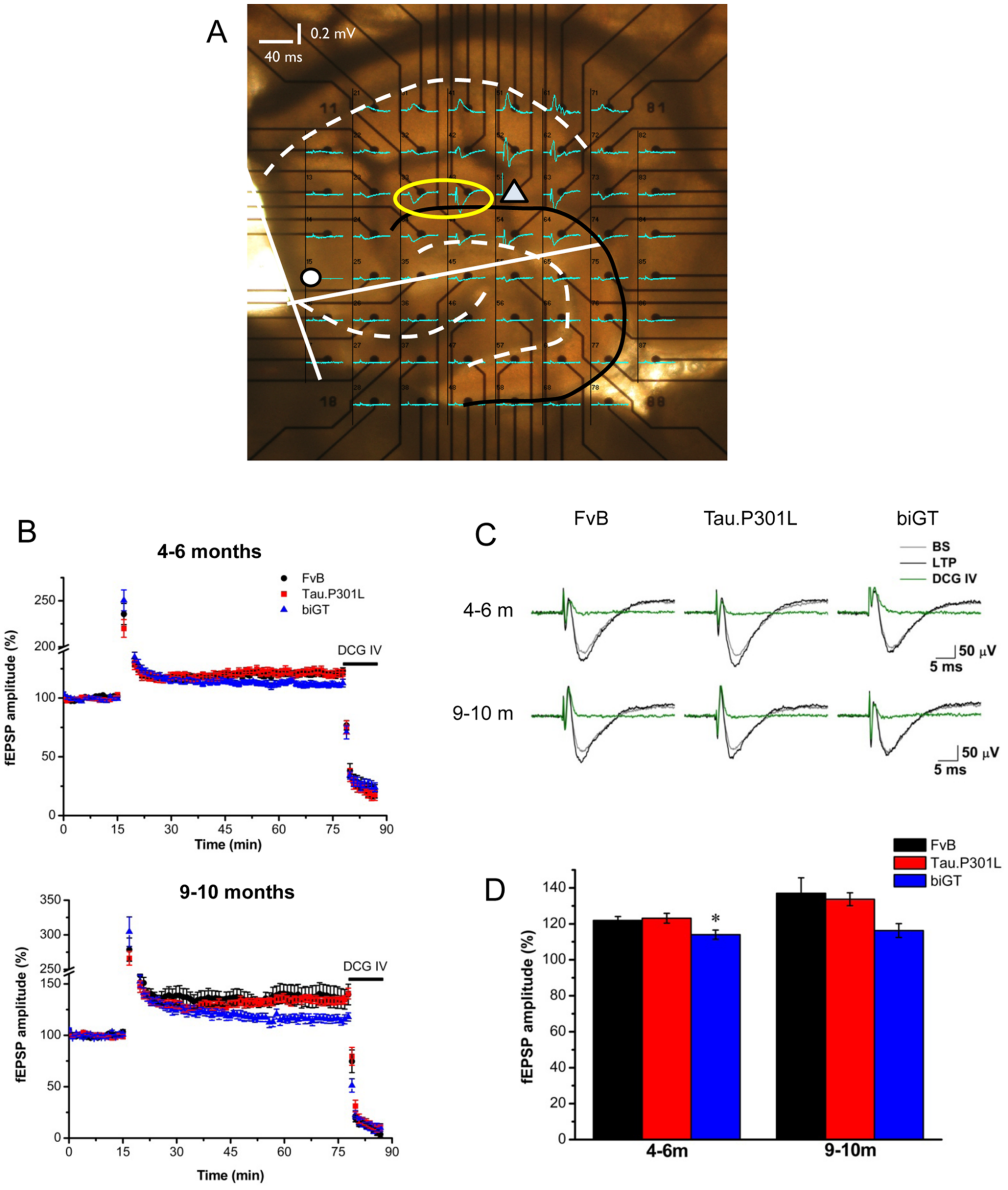
Electrophysiological analysis of LTP in SLM synapses in Tau.P301L and biGT mice. A. Image of hippocampal section on the MEA chip, with superimposed fEPSPs recorded in the SLM (yellow oval) in response to stimulation of the TA pathway (white triangle). White solid lines indicate where the section was cut to obtain pure TA responses. White dashed lines indicate granule and pyramidal cell body layers; black solid line indicates the hippocampal fissure. Electrode 15 (white circle) was used as internal ground electrode. B. fEPSPs tracings (mean±SEM) recorded in SLM in horizontal brain sections from young and old Tau.P301L and biGT mice. LTP was induced by 4 trains of high frequency stimulation (HFS) after 15 min of baseline recording. At the end of all recordings, DCG IV (3 µm) was added for 10 min. C. Representative tracings of fEPSPs at baseline (grey), at 60 min after HFS (black) and 10 min after DCG IV (green). D. fEPSPs amplitudes recorded between 30 and 60 min after HFS in young and old Tau.P301L and biGT mice (mean±SEM). Number of sections and mice: 4–6 months: FvB, n = 12 sections/8 mice; Tau.P301L, 11sections/6 mice; biGT 12 sections/6 mice; 9–10 months: FvB, n = 9 sections/6 mice; Tau.P301L 11 sections/6 mice; biGT, 8 sections/6 mice.

Synaptic activity in the SLM was examined ex vivo in brain sections of two age-groups of Tau.P301L and biGT mice, respectively 4–6 and 9–10 months, and compared to wild-type FvB mice, matched for age and gender and with the identical genetic background. Evoked fEPSPs were recorded in the SLM of CA1 by stimulating ERC efferent axons (Fig. 5A). Responses at SLM synapses were recorded without contamination from the SC synapses by surgical sectioning the SC CA1 pathway. Stimulation of the TA pathway evoked negative (sink) and positive (source) fEPSPs in SLM and SR, respectively [41], [42], [65]. Selective blocking of excitatory transmission at TA synapses with DCG IV, a specific mGluR2 agonist [42], [43] reduced nearly completely the evoked fEPSPs, confirming that TA responses were recorded.

**Figure 6:**
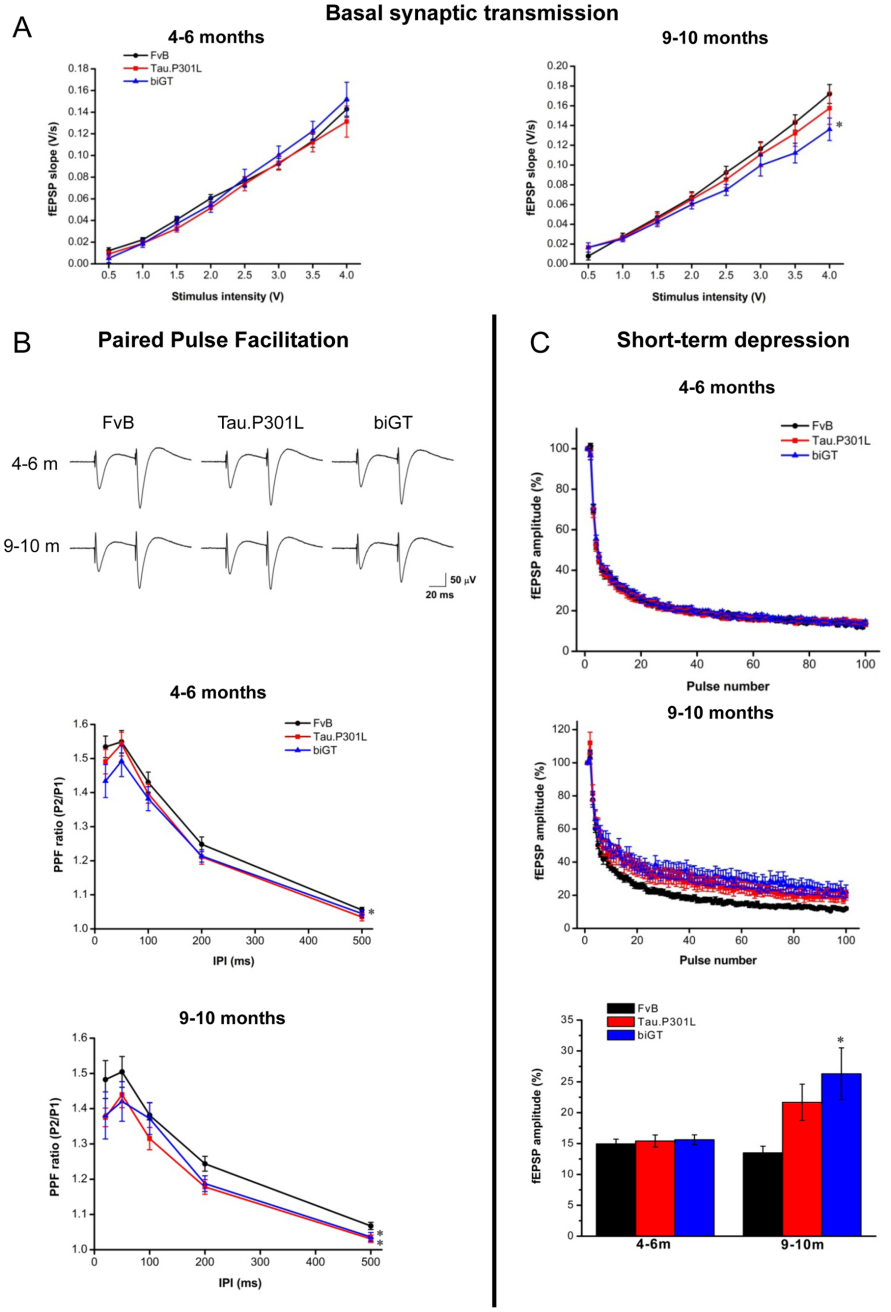
Electrophysiological analysis of SLM synapses in Tau.P301L and biGT mice. A. Basal synaptic transmission measured as the initial slope of fEPSPs in response to increasing stimulus intensities in young (left panel) and old mice (right panel). Old biGT mice differed significantly from wild-type FvB mice: *p = 0.005. B. Paired-pulse facilitation are expressed as amplitude ratio of second to first peak. Tracings are averaged at 50 ms IPI in young and old FvB, Tau.P301L and biGT mice. Statistical analysis at 4–6 months: *p = 0.021 biGT vs FvB; at 9–10 months: *p = 0.039 biGT vs FvB and p = 0.004 Tau.P301L vs FvB.

We measured LTP using our standard protocol of stimulation: 4 trains of 100 Hz pulses of 1 sec each, separated 20 sec apart. We successfully induced LTP in all three genotypes, with the expected amplitude at SLM synapses [41]. LTP at SLM synapses in young and old Tau.P301L mice was similar to those in age-matched wild-type FvB mice (Fig. 5B). Conversely, LTP was significantly reduced in SLM of young biGT mice (Fig. 5B,D). In old biGT mice, the LTP at SLM synapses was also decreased but did not reach statistical significance because of the known inherent greater variation of electrophysiological parameters in sections from old mice (Fig. 6B, lower panel).

Basal synaptic transmission was measured by compilation of Input/Output (I/O) curves with stimulus strengths between 0.5 and 4 V. In young mice, the I/O data in both genotypes were similar to wild-type mice (Fig. 6A, left panel). In old biGT mice (age 9–10 months) the fEPSP initial slopes were significantly decreased, whereas again, old Tau.P301L mice did not differ from age-matched wild-type mice (Fig. 6A, right panel).

Interesting in this context was the observation that in about 40% of old biGT mice, fEPSP could not be recorded in hippocampal subfields SLM, SR and DG because the amplitudes were below the detection limit set at peak-to-peak amplitude <50 µV using 2V stimuli for 200 µs (Table 2). The biGT mice clearly displayed an age-dependent disruption of basal activity at the TA synapses, but also at the Schaffer collaterals and at PP dependent synapses.

**Table 2:** Electrophysiological parameters.

	FvB		Tau.P301L		biGT	
Age (months)	4–6	9–10	4–6	9–10	4–6	9–10
success ratio (recorded/attempts)	12/12	9/9	11/11	11/11	12/12	8/14
number of mice	8	6	6	6	6	6
success-rate (%)	100	100	100	100	100	57

Short-term regulation of neurotransmitter release was measured by paired-pulse facilitation (PPF) and short-term depression at TA synapses. In young biGT mice, but not in young Tau.P301L mice, PPF was significantly reduced, while in old mice (age 9–10 months) the TA synapses showed reduced facilitation in both Tau.P301L and biGT mice, compared to wild-type mice (Fig. 6B).

Short-term depression was readily observed at TA synapses, induced by 100 Hz pulses for 1 sec. In young mice (4–6 months), short-term depression was not affected by either genotype, while in older mice (9–10 months) short-term depression was less pronounced in biGT mice (Fig. 6C).

Combined, the results demonstrated that Tau.P301L mice showed normal baseline transmission and normal LTP, but displayed reduced PPF at the SLM synapses. In contrast, synapses in the SLM of biGT mice were deficient in base-line transmission, and in short-term and long-term plasticity.

### Analysis of white matter and myelinated axons

It was obvious from the current detailed analysis of ERC and CA1 SLM that phosphorylation of protein tau was increased as early as age 3 months in both models. Moreover, age-dependent aggravation of tauopathy was demonstrated in the tri-synaptic pathway, essential for learning and memory. Of note, late hippocampal pathology, defined by IHC with AT100, was far more extensive in biGT mice than in Tau.P301L mice [27], [50], which demonstrates the essential contribution of GSK3β, although the molecular details and sub-regional differences remain to be defined. After we defined by AAV-Tau injection in the ERC that protein Tau made use of both the TA and PP pathways to reach the hippocampal formation (Fig. S1), we went on to assess the effects of protein Tau alone and in combination with GSK3β on the white-matter tracts within the CA1 SLM in both pre-clinical tauopathy models.

#### Impaired diffusion of DiI from ERC to CA through myelinated pathways

The reported electrophysiological data, especially the affected paired pulse facilitation (PPF) strongly implied presynaptic changes in Tau.P301L mice, exacerbated by GSK3β in the biGT mice. In addition, Nectin-3, despite being claimed as a post-synaptic CAM, was reported to co-localize with myelinated axons, and therefore hypothesized to be essential for TA myelinated axons [66], [67]. Consequently, we investigated presynaptic parameters for alterations of myelinated axons of the TA pathway within CA1 SLM.

The carbocyanine dye DiI is the preferred tracer for long axonal tracts, despite the drawback of slow diffusion, because its distribution relies on lateral diffusion within the axonal membranes, also in fixed tissues. Moreover, DiI labels myelin sheaths over very long distances [68]. DiI crystals were implanted in the ERC in fixed brains from adult pre-symptomatic Tau.P301L mice (age 6–7 months) and age-matched wild-type FvB mice. Brains were analyzed histologically after 14 months of incubation.

**Figure 7:**
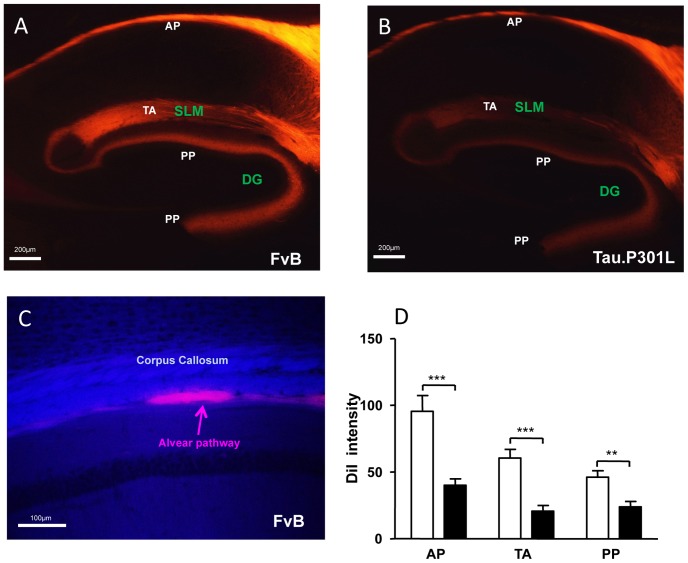
DiI diffusion is reduced in brain of Tau.P301L mice. A,B: images of DiI diffusion patterns from implants in the ERC of brains from FvB (A) and Tau.P301L mice (B) after 14 months incubation. These are representative confocal images but with strongly different contrast and enhancement for the Tau.P301L mice (+3 gain, +4 exposure) which was needed to visualize the pathways. C. Double histological staining of DiI pattern combined with CMC staining for myelin (blue) to locate the alvear pathway (AP) relative to the corpus callosum (CC). D. DiI staining intensity in indicated brain regions of FvB (n = 8) and Tau.P301L mice (n = 8). Statistical analysis: Student’s t-test (unpaired, two-tailed). **p<0.01 and ***p<0.001.

In wild-type mouse brain, the PP and TA were easily identified by the bright staining of their axons (Fig. 7A). In contrast, in brain from Tau.P301L mice, the intensity of staining of both TA and PP axonal pathways was significantly reduced compared to wild-type mice (Fig. 7). Also the alvear pathway (denoted AP in Fig. 7) located below the corpus callosum (CC) was brightly stained in wild-type mice, and significantly less in the brain of Tau.P301L mice (Fig. 7C,D). Interestingly, both AP and TA originate in ERC layer III and were observed to be similarly defective for DiI diffusion, while PP was affected as well although originating in ERC layer II. Obviously, all the major ERC-CA connections were structurally and functionally compromised by neuronal expression of protein Tau.P301L.

#### Defective myelination of TA axons in Tau.P301L, exacerbated in biGT mice

The SLM comprises the projection of myelinated axons in the TA pathway that originate in the ERC and synapse onto CA1 pyramidal neurons. Because myelin sheaths are easily recognized ultrastructurally, we observed during the ultrastructural analysis of spines, described in a foregoing section, that myelin structures of TA axons in Tau.P301L mice appeared damaged, already at the very young age of 4 months. Surprisingly, the ultra-structure images showed many defective myelin sheaths, referring to similar observations first in the brain of aged primates, later also in elderly humans and AD patients [2], [69].

**Figure 8:**
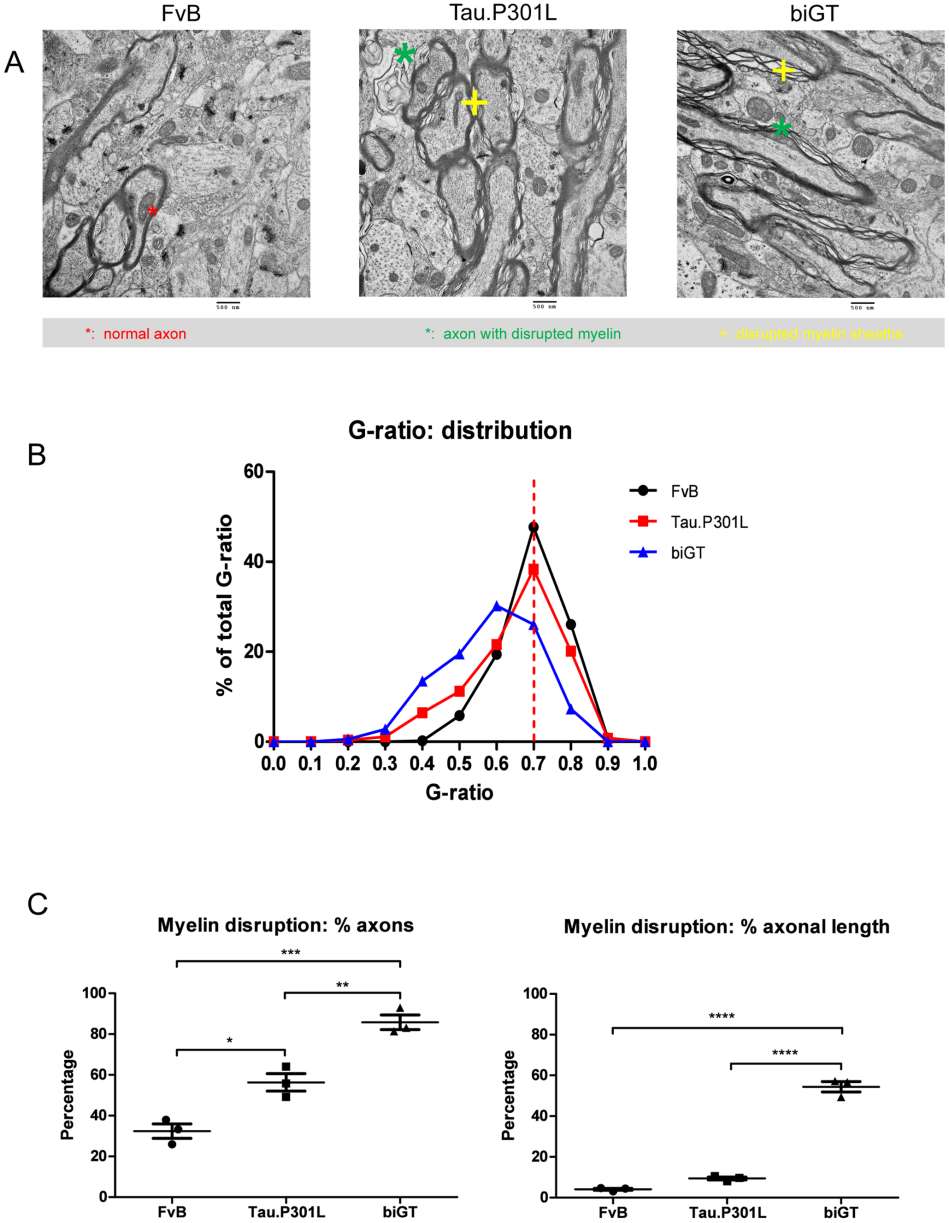
Myelin defects in SLM of Tau.P301L mice. A. Representative images of the ultrastructure of myelinated axons in wild-type, Tau.P301L and biGT mice, with myelin defects indicated by the symbols explained in the caption. B. G-ratio distribution of TA axons in SLM in wild-type FvB, Tau.P301L and biGT mice (see also Table 2). C. Quantitation of axons harboring morphological defects (left panel) and of axonal length with disrupted myelin sheaths (right panel) in TA axons of SLM in the three genotypes. Data are mean±SEM, statistically analyzed by one-way ANOVA followed by Bonferroni Multiple Comparison Test post hoc test; *p<0.05, **p<0.01, ***p<0.001.

Operationally, we here defined myelin defects as sections of axons surrounded by myelin sheaths that were malformed, uncompacted, and presenting with split lamellae (Fig. 8A). The G-ratio, defined as the ratio of the diameter of axon to that of the total myelinated fiber, is the preferred parameter of myelinated axons, with a normal value of around 0.77 in CNS of mice [70]. In Tau.P301L mice, the G-ratio of TA axons was distributed around the median of 0.70, with a small shift in distribution to lower values compared to wild-type FvB mice (Fig. 8B; Table 3). In contrast, in the SLM of biGT mice a significant deviation from the mean G-ratio, with the median value decreased to 0.59, while moreover the distribution was strongly skewed to lower values (Fig. 8B, Table 3).

**Table 3:** G - ratio of TA axons.

	wild-type FvB		Tau.P301L		biGT	
	n	%	n	%	n	%
O<G-ratio <1	499	100	527	100	533	100
G-ratio <0.7	243	48.7	309	58.6	443	83.1
G-ratio >0.7	256	51.3	218	41.4	90	16.9

Additional parameters and means of quantification of the observed myelin defects were applied to the TA axons in the SLM. We first measured the relative number of TA axons that showed myelin disruptions, which indirectly confirmed the data of the G-ratio for the biGT mice but not for the Tau.P301L mice. Indeed, despite the normal G-ratio observed in Tau.P301L mice, a significant number of TA axons in the SLM were damaged with respect to myelin structure compared to FVB mice (Fig. 8C, left panel). Moreover, in biGT mice nearly all axons in the SLM were covered by damaged myelin sheaths (85.8±3.6%; One-way ANOVA: F_(2,6)_ = 49.05, p = 0.0002; Fig. 8C, left panel).

The final parameter assessed for myelin defects in the SLM, the relative length of disrupted axonal myelin sheaths indicated a trend to increased length of damaged axons in Tau.P301L mice (∼10% of their length). Conversely, in biGT mice more than 50% of the total length of the myelinated axons in the SLM contained damaged myelin sheaths (One-way ANOVA: F_(2,6)_ = 320.6, p<0.0001; Fig. 8C, right panel).

#### CNPase as marker of defective myelinated TA axons in Tau.P301L mice and biGT mice

We went on to molecularly characterize the myelin defects in both transgenic models by biochemical and immunohistological analysis of CNPase, a myelin-associated enzyme that is among the first expressed in differentiating oligodendrocytes that are responsible for myelination of axons in the CNS [71], [72].

**Figure 9:**
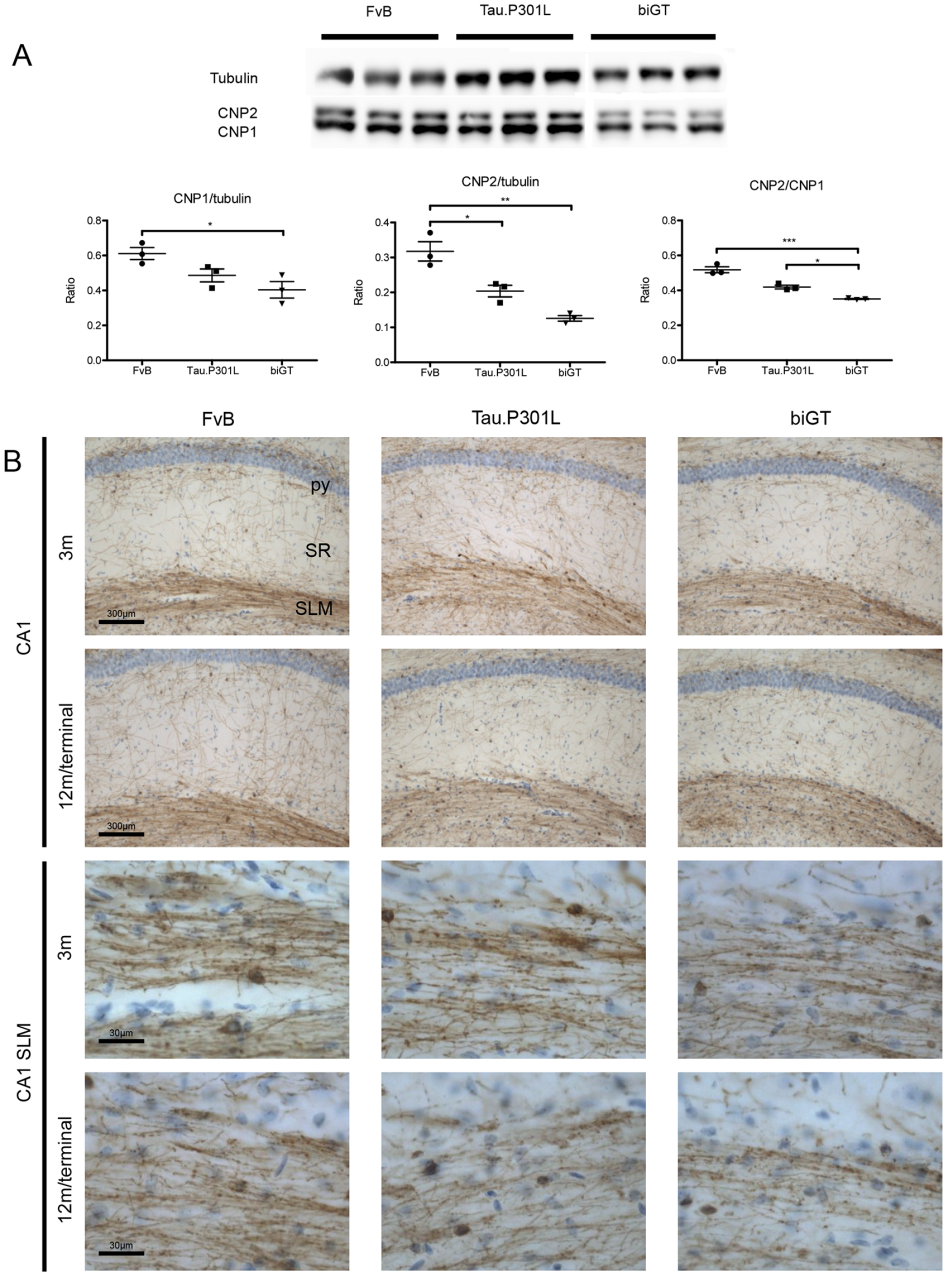
Analysis of oligodendrocyte marker CNPase. A. Representative western for CNPase in hippocampal protein extracts from wild-type FvB, Tau.P301L and biGT mice (n = 3 per genotype). The two CNPase isoforms are marked CNP1 and CNP2. B. Representative IHC images for CNPase in SLM of the three genotypes, at ages indicated in the captions. CA1 hippocampal sub-regions of interest are defined as stratum pyramidale (py), radiatum (SR) and stratum lacunosum molecular (SLM).

Biochemically, the two known isoforms of CNPase were investigated and quantified by western blotting (Fig. 9A). In Tau.P301L mice the levels of CNP2 were significantly decreased, while CNP1 also tended to lower levels (Fig. 9A). In adult biGT mice (age 6 months) the hippocampal levels of both the isoforms of CNPase were significantly reduced (Fig. 9A). The ratio of the levels of both CNPase isoforms was significantly lower in the hippocampus of both Tau.P301L and biGT mice (Fig. 9A, right panel).

IHC for CNPase in brain sections of young, old and terminal mice of both genotypes, compared to wild-type FvB mice, visualized the perturbations of myelin by weaker immunostaining in the CA1 SLM of Tau.P301L and biGT mice (Fig. 9B). CNPase immunoreactivity was mainly confined to the myelinated axons within the SLM, implicating that the decrease in CNPase isoforms detected biochemically, was contributed primordially by SLM axons. Technically, it proved not possible to reliably quantify the CNPase immunostaining to assign the decreased CNPase is in the number of myelinated fibers or in their deficient myelination, although the overall number of axons was not affected. Conversely, the defective CNPase levels corroborated the DiI diffusion data and the myelin defects, presented in the foregoing sections.

The combined outcome illustrated not only the stronger effect of the early stages of beginning tauopathy on the CNP2 isoform, but also the synergistic impact of GSK3β with Tau.P301L on axonal myelination in the hippocampus of the bigenic biGT mice, with inherently higher GSK3β activity. The combined data-sets demonstrated that myelination of axons within the TA was already altered at young age in Tau.P301L mice, and that this clinical phenotype was dramatically aggravated by co-expression of GSK3β with Tau.P301L in the biGT mice.

## Discussion

Indications that tauopathy, and pathological components of AD, originate in the trans-entorhinal cortex were proposed long ago [11]. To unravel the elusive molecular mechanisms that act early in sporadic AD, we need to define and understand the temporal and spatial origin and progression of the molecular changes in amyloid and protein tau and their associated pathology. Mouse models are most informative to define parameters that with current technologies are impossible to address in humans.

### Protein Tau and the ERC-SLM-CA1 connection

Here we addressed the functional and structural problems caused by tau pathology in the entorhinal cortex and hippocampus in our two validated tauopathy models [25]–[27], [46], [49], [51], [52]. We established that increased phosphorylation of protein tau was evident in neurons in the medial and lateral entorhinal cortex. Interestingly, the lateral ERC layer III was affected at younger age than the medial ERC, as observed in patients [12]. The observations were extended from the Tau.P301L mice to the bigenic biGT mice that contain the same homozygous Tau.P301L transgenic make-up, with the extra addition of one allele of the GSK3β.S9A transgene [25], [27], [73].

Phosphorylation of protein Tau was prominent in the SLM in both models, which led us to investigate the TA and PP pathways that connect ERC and CA1 SLM, deriving from our observation of specific reduction of the synaptic CAM Nectin-3 in the SLM of these models [25]. Additionally, we now highlighted by unilateral injection of AAV-Tau.4R in the ERC, the primary connections of interest in the PP and TA pathways leading from ERC to the hippocampus proper (Fig. S1). Our observations, combined with literature data discussed in the next sections, demonstrated that increased phosphorylation of protein Tau, as prelude of or for beginning tauopathy, inflicted important functional and structural defects within the CA1 SLM region.

The TA pathway has been overlooked in studies of the hippocampal formation, its wiring and functions. Its physiological importance was enforced by complex genetic manipulation in mice to specifically and reversibly inactivate synapses in CA1 formed by TA axons originating in ERC layer III [20], [74].

The pathological contributions of a functional ERC-CA connection to the temporal and spatial progression of AD was corroborated recently in mice expressing mutant APP or protein Tau in ERC layers II/III [13], [75]–[77]. The comparable Tau.P301L model failed to show behavioral defects, although tauopathy was induced in the projection areas of the ERC [76]. Two more models expressing Tau.P301L in the ERC supported the hypothesis that tauopathy progresses from the ERC to the hippocampus what was attributed to the PP, which does originate in the ERC layer II but targets mainly the DG [13], [77].

### Protein Tau and cognitive parameters of SLM-CA1

The various defects in the tauopathy models are substantiated by electrophysiological and behavioral analysis. The defective cognition of young Tau.P301L mice was extended to later age when still preclinical with respect to progressive motor impairment incurring at older age. The electrophysiological analysis ex vivo, corroborated the performance of in vivo tasks that defined the hippocampal cognitive impairment of Tau.P301L mice.

We recently described impaired levels of Nectin-3 in CA1 SLM in Tau.P301L mice, already at young age [25]. Combined with the timeline of cognitive defects described here, the data demonstrate that defective Nectin-3 levels correlate with defective hippocampal/ERC based learning and memory. Interestingly, most recent knock-down of hippocampal Nectin-3 caused loss of dendritic spines and spatial memory defects [78]. The combined data underpin the important contribution of Nectin-3 in memory processing, and the negative effects of tau pathology, even in its most early stages of augmented phosphorylation at young age. The latter was recently observed in post-mortem human brain of young adults and even children [79].

### Spines, synapses and Myelin affected by protein tau and GSK3β

Needless to state that integrity of myelin sheaths is essential for proper axonal functioning. The defective synaptic transmission at TA synapses in the SLM can be contributed at least in part to myelin defects, further corroborated by the aggravating contribution of GSK3β. The biGT mice at any age produce weaker LTP than the Tau.P301L mice, indicating that postsynaptic influx of Ca^2+^ through NMDA receptors and voltage-gated L-type Ca^2+^ channels can be affected [80], [81]. The data imply that protein Tau and GSK3β affect pre- and post-synaptic functions and mechanisms of synaptic signal transmission.

Phosphorylated protein Tau did not affect dendritic spine density in the SLM in young mice (4–6 months), in contrast with the surprising increased spine density in other hippocampal sub-regions of Tau.P301L mice [29]. Nevertheless, ultrastructure of mushroom spines was affected in the SLM of clinically pre-symptomatic Tau.P301L mice that were cognitively impaired [26,27,29,52; this study]. The concomitant increase in PSD volume and area in Tau.P301L mice is to be regarded as a compensatory effect that is reminiscent of the increased PSD95 levels in patients [56]. Of note, thinning of the SLM and stratum radiatum is correlated with cognitive decline in humans [82]. In addition, the combination of ERC and hippocampal Tau pathology, as well as that within the TA track, paralleled the early cognitive decline in behavioral tasks, although to our knowledge, no behavioral task specifically relies on the TA pathway or on the SLM.

### Defective Myelinated axons and Tauopathy

AD is by definition a neurodegenerative disease striking gray matter. White matter pathology is rarely discussed, but comprises myelin defects and demyelination, along with axonal degeneration [2]–[4], [7]–[9], [61]. Defects were reported in myelinated PP axons [7], [10], while white matter microstructure was closely associated with levels of protein Tau and Aβ42 in CSF [83]. In animal models, myelin defects were observed in the cortex of tau mice [84], while combined amyloid and tau pathology caused minor myelin defects of axons denoted as Schaffer collaterals that connect CA3 to CA1 [85]. As a caveat, one must note that in rodents the Schaffer collaterals are not myelinated [86]–[90]. Only very recently were myelin defects described in an amyloid mouse model [91]. The combined data indicate that myelinated axons are affected early by amyloid but particularly by protein Tau, before the classical AD pathology is evident [92].

Our observation of altered PPF, indicative for presynaptic defects and therefore axonal signal disruption, as well as the potential contribution of Nectin-3 to myelination [66], [67], led us to examine ultrastructurally the TA axons. Our data corroborates and considerably extend the records of myelin defects in AD-models.

Neuronal protein Tau damaged myelinated TA axons, with dramatic aggravation by the neuronal co-expression of GSK3β. This unexpected extra axonal damage by increased phosphorylation of protein Tau by GSK3β, is proposed to disable surrounding oligodendrocytes in maintaining myelination of TA axons in biGT mice. The underlying mechanisms are to be found in the complex interplay of axons and oligodendrocytes, which remain largely elusive and are intensely investigated in demyelinating diseases. The prime interest is in multiple sclerosis, in which a recent provocative hypothesis poses that degenerating axons induce damage of surrounding myelin, not the other way around [93]. Our data on CNPase corroborate the structural defects of myelin sheaths, in line with decreased CNPase activity in the hippocampus in AD [94], [95]. Also in this framework fits the remarkable correlation of CSF biomarkers with white matter defects in AD, promoting interest in white matter disruption as a diagnostic criterion in AD, as measured by MRI diffusion tensor imaging [9], [82], [83], [96].

The combined data-sets demonstrate that increased levels of protein Tau are sufficient to profoundly impair the functional and structural organization of the entorhinal - hippocampal complex. In particular the synapses and axons in the stratum lacunosum moleculare were concerned, which engendered severe white matter pathology along the myelinated axons of the transammonic pathway. The severe extra negative impact on the white matter defects by GSK3 implies a direct relation of axonal damage to the functioning of surrounding oligodendrocytes, stemming from increased phosphorylation of neuronal protein Tau.

## Supporting Information

Figure S1 **Intracerebral injection of AAV-Tau.4R in ERC highlights TA and PP pathways.** Unilateral injection of AAV-Tau.4R in the ERC of wild-type FvB mice was analyzed 14 days post injection by IHC for human Tau with HT7 on coronal brain sections. A. Representative images of hippocampal formation and ERC in ipsi- and contra-lateral hemispheres. The middle panels are enlarged views of the boxed areas in the left panels to illustrate neuronal expression of human Tau in the ERC and CA1 sub-regions. B. Enlarged view of the relevant strata: SR, stratum radiatum; SLM, stratum lacunosum moleculare; ml, molecular layer; PP, perforant pathway; gl, granular layer; hilus of the gyrus dendatus.
(TIF)
