# Supplementary figures and images for: Early Structural and Functional Defects in Synapses and Myelinated Axons in Stratum Lacunosum Moleculare in Two Preclinical Models for Tauopathy
Source: PLoS One. 2014 Feb 3;9(2):e87605. doi: 10.1371/journal.pone.0087605 (PMC3912020; doi:10.1371/journal.pone.0087605)

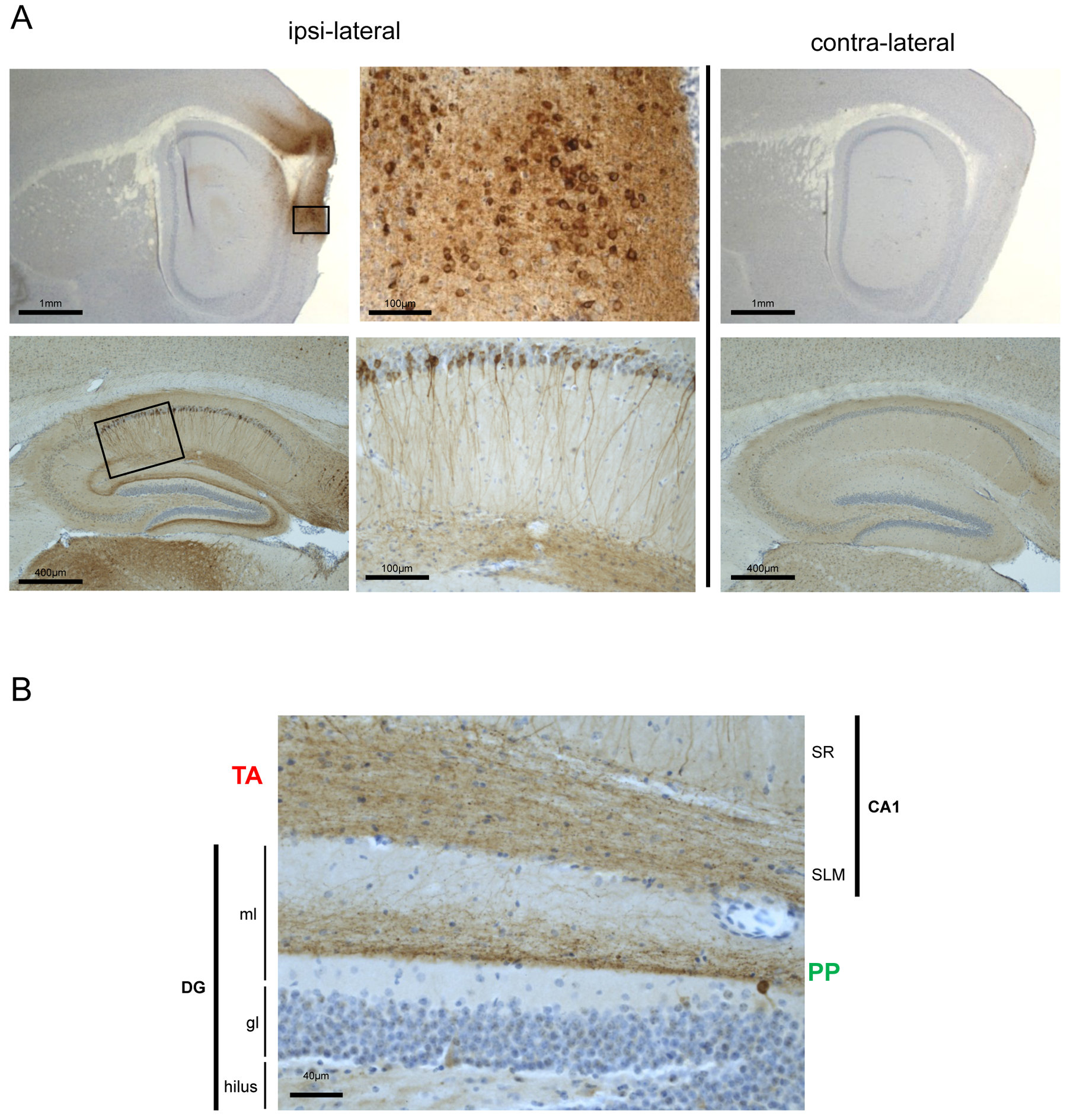

Supplement: Figure S1 — Intracerebral injection of AAV-Tau.4R in ERC highlights TA and PP pathways. Unilateral injection of AAV-Tau.4R in the ERC of wild-type FvB mice was analyzed 14 days post injection by IHC for human Tau with HT7 on coronal brain sections. A. Representative images of hippocampal formation and ERC in ipsi- and contra-lateral hemispheres. The middle panels are enlarged views of the boxed areas in the left panels to illustrate neuronal expression of human Tau in the ERC and CA1 sub-regions. B. Enlarged view of the relevant strata: SR, stratum radiatum; SLM, stratum lacunosum moleculare; ml, molecular layer; PP, perforant pathway; gl, granular layer; hilus of the gyrus dendatus. (TIF) [file pone.0087605.s001.tif]
